# A simulation study: comparing independent component analysis and signal-space projection – source-informed reconstruction for rejecting muscle artifacts evoked by transcranial magnetic stimulation

**DOI:** 10.3389/fnhum.2024.1324958

**Published:** 2024-05-09

**Authors:** Tuomas Petteri Mutanen, Ida Ilmoniemi, Iiris Atti, Johanna Metsomaa, Risto Juhani Ilmoniemi

**Affiliations:** Department of Neuroscience and Biomedical Engineering, Aalto University School of Science, Espoo, Finland

**Keywords:** transcranial magnetic stimulation, electroencephalography, data analysis, artifact rejection, independent component analysis, signal-space projection, modelling, simulation

## Abstract

**Introduction:**

The combination of transcranial magnetic stimulation (TMS) and electroencephalography (EEG) allows researchers to explore cortico-cortical connections. To study effective connections, the first few tens of milliseconds of the TMS-evoked potentials are the most critical. Yet, TMS-evoked artifacts complicate the interpretation of early-latency data. Data-processing strategies like independent component analysis (ICA) and the combined signal-space projection–source-informed reconstruction approach (SSP–SIR) are designed to mitigate artifacts, but their objective assessment is challenging because the true neuronal EEG responses under large-amplitude artifacts are generally unknown. Through simulations, we quantified how the spatiotemporal properties of the artifacts affect the cleaning performances of ICA and SSP–SIR.

**Methods:**

We simulated TMS-induced muscle artifacts and superposed them on pre-processed TMS–EEG data, serving as the ground truth. The simulated muscle artifacts were varied both in terms of their topography and temporal profiles. The signals were then cleaned using ICA and SSP–SIR, and subsequent comparisons were made with the ground truth data.

**Results:**

ICA performed better when the artifact time courses were highly variable across the trials, whereas the effectiveness of SSP–SIR depended on the congruence between the artifact and neuronal topographies, with the performance of SSP–SIR being better when difference between topographies was larger. Overall, SSP–SIR performed better than ICA across the tested conditions. Based on these simulations, SSP–SIR appears to be more effective in suppressing TMS-evoked muscle artifacts. These artifacts are shown to be highly time-locked to the TMS pulse and manifest in topographies that differ substantially from the patterns of neuronal potentials.

**Discussion:**

Selecting between ICA and SSP–SIR should be guided by the characteristics of the artifacts. SSP–SIR might be better equipped for suppressing time-locked artifacts, provided that their topographies are sufficiently different from the neuronal potential patterns of interest, and that the SSP–SIR algorithm can successfully find those artifact topographies from the high-pass-filtered data. ICA remains a powerful tool for rejecting artifacts that are not strongly time locked to the TMS pulse.

## Introduction

Transcranial magnetic stimulation (TMS) is a technique that allows the safe and non-invasive activation of the human cortex (Barker et al., 1985). A strong current pulse is passed through a stimulating coil, positioned above the cortical region of interest. This current pulse immediately generates a time-varying magnetic field around the coil, which in turn induces an electric field within the brain. With strong enough stimulation intensity, the induced electric field depolarizes neurons sufficiently to cause them to fire action potentials (Ilmoniemi et al., 1999). The TMS-generated cortical activity can be measured directly with concurrent electroencephalography (EEG) recordings (Ilmoniemi and Kičić, 2010; Tremblay et al., 2019; Hernandez-Pavon et al., 2023). However, the EEG data are often compromised by TMS-evoked muscle artifacts; the induced electric field can activate scalp muscles resulting in short-lived but high-amplitude artifact signals right after the TMS pulse (Mutanen et al., 2013).

TMS-evoked muscle artifacts can be minimized during recordings to an extent by optimizing the location and orientation of the TMS coil (Mutanen et al., 2013). However, when targeting certain regions of interest, such as Broca’s area, the online minimization is not feasible due to the proximity of the scalp muscles (Korhonen et al., 2011; Mutanen et al., 2013). Korhonen et al. (2011) tested independent component analysis (ICA) for identifying and removing the muscle artifacts. The ICA methodology has been further elaborated for this application in many later articles (Hernandez-Pavon et al., 2012, 2022; Metsomaa et al., 2014; Rogasch et al., 2014).

ICA can be mathematically explained using the standard linear model for measured EEG data (Eq. 1). The measured data Y can be written in terms of the brain signals $Y_{\mathrm{brain}}$ , artifact signals $Y_{\mathrm{art}}$ , and noise N .


(1) $$Y=Y_{\mathrm{brain}}+Y_{\mathrm{art}}+N$$

With ICA, the measured data can be decomposed into statistically independent components:


(2) Y=AS,

where A is the mixing matrix, holding in its columns the potential patterns (or topographies) of the underlying independent components, and S holds the time courses of the independent components on its rows. If we can recognize which components reflect mostly artifacts in Eq. 2, we can separate the mixing matrix into brain and artifact mixing matrices, $A_{\mathrm{brain}}$ and $A_{\mathrm{art}}$ , respectively, and simply clean the data as follows:


(3) $$\begin{array}{l} Y=A_{\mathrm{brain}}S_{\mathrm{brain}}+A_{\mathrm{art}}S_{\mathrm{art}}+A_{\mathrm{noise}}S_{\mathrm{noise}},\\ \;\Rightarrow Y_{\mathrm{brain}}\approx Y-A_{\mathrm{art}}S_{\mathrm{art}}\text{.} \end{array}$$

Note that we have omitted noise N from the further discussion (Eq. 3). With noise, we refer to longer-lasting, relatively stationary signal disturbances, such as line noise at 50/60 Hz or thermal noise (or Nyquist noise), whereas artifacts are typically shorter-lasting signal deflections. Noise could be either cancelled by removing such independent component that reflect noise ( $A_{\mathrm{noise}}S_{\mathrm{noise}}$ ), or using other standard preprocessing steps, such as Fourier filtering, if the frequency spectra of noise and neuronal signals do not overlap considerably.

ICA is a powerful technique for unmixing multidimensional data into a set of components if the latent components are truly independent. However, this assumption is most likely violated (Metsomaa et al., 2014) in TMS-evoked EEG potentials (TEPs) (Paus et al., 2001; Lioumis et al., 2009) as both the muscle artifacts and direct transcranial reactions to the electric field are time locked to the same TMS pulse.

Mäki and Ilmoniemi (2011) introduced an alternative approach to suppress TMS-evoked muscle artifacts with signal-space projection (SSP) (Uusitalo and Ilmoniemi, 1997). SSP projects out those topographies from the EEG data that are likely to reflect artifacts. Mäki and Ilmoniemi (2011) estimated muscle artifacts topographies by taking the largest singular vectors of high-pass filtered data (cut off 100 Hz, see Eqs. 4–6). This route could be taken, assuming that neuronal EEG signals are mostly manifested at frequencies clearly below 100 Hz (Buzsaki and Draguhn, 2004) but electromyography signals show a broadband response. Thus, the high-pass filtered data should mainly consist of artifact topographies, rather than neuronal ones. After estimating the muscle-artifact topographies, an SSP operator was formed (Eq. 7). This operator projects the data onto a signal space orthogonal to the artifact topographies. While SSP effectively suppressed artifact signals, it also introduced distortions to the EEG-channel signals, thus complicating the physiological interpretation of the data (Mäki and Ilmoniemi, 2011). The reason for these distortions is best explained by Eqs. 9, 10; the original lead-field matrix **L**, which describes how neuronal activity is mixed in the EEG channel-space, is modified by the SSP-operator **P**. Mutanen et al. (2016) introduced an additional step called source-informed reconstruction (SIR) to mitigate these signals distortions and to facilitate the interpretation of the artifact-cleaned EEG signals.

The SSP–SIR approach is summarized in Eqs. 4–12. By high-pass filtering the data in Eq. 1 from 100 Hz, most of the high-passed data, $H\left(Y\right)$ , consist of artifacts and noise (Eqs. 4, 5).


(4) $$H\left(Y\right)=H\left(Y_{\mathrm{brain}}\right)+H\left(Y_{\mathrm{art}}\right)+H\left(N\right)$$

(5) $$H\left(Y\right)\approx H\left(Y_{\mathrm{art}}\right)+H\left(N\right)$$

If we compute the singular value decomposition of the high-passed data (Eq. 6), we can form a spatial filtering operator P (Eq. 7) that projects out the *k* most prominent artifact dimensions from the original data Y (Eq. 8).


(6) $$H\left(Y\right)=U\Sigma V^{T}$$

(7) $$P=I-U_{1:k}U_{1:k}^{T}$$

(8) $$PY\approx PY_{\mathrm{brain}}+PN$$

Even if the spatial filter can remove artifact signals effectively from the data ( $PY_{\mathrm{art}}\approx 0$ ) we can see from Eq. 8 that the neuronal signals are distorted with the matrix P . However, we can take this into account in the inverse estimation. If we write **Y**_brain_ in terms of the lead field **L** and the unknown brain activity **X**, generating the neuronal EEG signals


(9) $$Y_{\mathrm{brain}}=LX$$

we can combine Eqs. 8, 9 to get:


(10) PY=PLX+PN.

Again, omitting the remaining noise PN , which could be suppressed using subsequent preprocessing steps if necessary, we estimate the unknown neuronal activity X that produces the artifact-suppressed EEG signals by taking the pseudoinverse of **PL**:


(11) $$\hat{X}=\left(\mathrm{PL}\right)†PY\text{.}$$

There are several ways to form the pseudoinverse ${\left(\mathrm{PL}\right)}^{†}$ . One option is using truncated singular value decomposition as in Mutanen et al. (2016). We can compute the final estimate for the brain EEG signals as follows:


(12) $$Y_{\mathrm{brain}}\approx L\left(\mathrm{PL}\right)†PY$$

The reconstruction of original brain signals $Y_{\mathrm{brain}}$ using Eq. 12 is called source-informed reconstruction (SIR) (Mutanen et al., 2016). The combined SSP–SIR approach is a spatial filtering method, which, unlike ICA, is not sensitive to the temporal correlation between the rejected artifact and the neuronal time courses, provided that spatial filter, $L{\left(\mathrm{PL}\right)}^{†}P$ in Eq. 12, has been successfully estimated. Instead, already the original SSP publication showed that the success of SSP depends on the dissimilarity of the topographies to be projected out and the neuronal topographies that we want to preserve (Uusitalo and Ilmoniemi, 1997).

Since the introduction of ICA and SSP–SIR approaches, various research groups have adopted them. See, e.g., (Rocchi et al., 2018; Darmani et al., 2019; Belardinelli et al., 2021; Luo et al., 2023) for ICA and (Fernandez et al., 2021; Zazio et al., 2021; Bracco et al., 2023; Mosayebi-Samani et al., 2023) for SSP–SIR. However, it has been an open question how the performance of these alternative strategies compares with one another. In a recent study, Bertazzoli et al., 2021 showed that the cleaning outcome depends on the selected methods. However, in the absence of a ground truth signal, it is impossible to conclude which of the preprocessed EEG signals better correspond the true underlying neuronal signals. The aim of this work is to test through simulations how the spatiotemporal properties of the TMS-evoked scalp-muscle responses affect the artifact-suppression performance of ICA and SSP–SIR.

Here, we generated distinct TMS-evoked artifacts through simulation and subsequently overlaid them upon an authentic pre-processed TMS–EEG dataset, serving as the ground truth. We manipulated two principal artifact attributes: the artifact potential scalp patterns (or topographies) and the extent of inter-trial variability characterizing the trial-specific artifact time courses. The corrupted synthetic datasets were preprocessed using either the ICA or SSP–SIR technique. After the application of these correction methods, the preprocessed datasets and the reference ground truth were compared.

ICA is known to work well in cancelling ocular artifacts (Hernandez-Pavon et al., 2023). This can be explained by the sporadic nature of these artifacts; blinks and eye movements often occur at random instances across the trials, and thus, can be expected to be statistically relatively independent from the time-locked TMS-evoked activity. Hence, our hypothesis was that ICA would work better when the artifact time courses show greater inter-trial variability. In contrast, we expected SSP to be particularly sensitive to the topographical similarity between the artifacts and the neuronal ground truth data, but insensitive to the time courses and their inter-trial variability. Finally, we hypothesized that TMS-evoked muscle artifacts recorded in real-world settings would exhibit high time-locking to the TMS pulse and display topographies relatively incongruent with those of neuronal potential patterns.

## Materials and methods

### Measured TMS–EEG data

The TMS–EEG dataset, used in the simulations as a ground truth, consists of 173 accepted high-quality epochs, measured from a right-handed 24-year-old female. Prior to the experiment, the participant provided a written consent. The research protocol was approved by the Ethics Committee of the Hospital District of Helsinki and Uusimaa and conformed with the Declaration of Helsinki. The participant was comfortably seated and instructed to fixate her gaze upon a centrally placed cross on the wall. Biphasic TMS pulses were targeted to the right primary motor cortex using the navigated Nexstim eXimia system (Nexstim Oyj, Helsinki, Finland) and a figure-of-eight coil with an outer diameter of 70 mm. The single pulses were delivered at 90% of the motor threshold, defined as the weakest stimulation intensity producing at least 5/10 motor-evoked potentials in the left abductor pollicis brevis with peak-to-peak amplitude of at least 50 μV.

EEG recordings were collected via a scalp array of 60 electrodes in the 10–20 montage. A reference electrode was affixed to the right mastoid, and the ground electrode was positioned on the skin surface above the right cheekbone. Electrode impedances were maintained below 5 kΩ. Concurrent EEG was acquired through the TMS-compatible Nexstim eXimia EEG system (Nexstim Oyj, Helsinki, Finland), which uses sample-and-hold circuitry, and thus, does not measure the TMS-pulse artifact (Virtanen et al., 1999). The EEG amplifier applied band-pass filtering (0.1–350 Hz) to the analog voltage signals before the data were digitized at the 1,450 Hz sampling frequency.

To mitigate auditory artifacts resulting from the loud TMS-coil click (Nikouline et al., 1999; ter Braack et al., 2015; Rocchi et al., 2021), the participant was exposed to white noise throughout the experiment. The sound-pressure levels were calibrated to never exceed 90 dB, with the help of a calibrated phantom ear.

The neuronavigation, providing the spatial information for accurate TMS coil positioning, relied upon T1-weighted magnetic resonance imaging (MRI) scans. The MRI scans were captured employing a 1-mm MPRAGE sequence.

The collected data were pre-processed to clean any TMS-related noise and artifact signals to produce a clean set of TMS-evoked potentials that worked as a ground truth in the analysis. This specific dataset was chosen to serve as the ground truth, since the raw EEG signals suffered from very small TMS-evoked artifacts and the pre-processing could be kept minimal. The preprocessing consisted of the following 10 steps:

1. Data epoching. The continuous raw EEG signals were divided into windows surrounding the TMS pulses. The dataset was divided into 3-s segments containing 1.5 s before and after each pulse.
2. The average of a pre-stimulus period of −500 to −5 ms with respect to the TMS pulse was subtracted from the data to correct for any baseline shifts.
3. The signal from each EEG channel was visualized and channels with visibly poor data quality (T3, C5, P1, P2) were removed from further analysis.
4. The raw trials were visualized and epochs with visibly poor data quality were removed (19 in total).
5. Each trial was detrended to reject slow drift, using robust detrending and third order polynomial model (de Cheveigné and Arzounian, 2018).
6. The dataset was decomposed into independent components using the FastICA algorithm (Hyvärinen and Oja, 2000). We rejected two components corresponding to the lateral ocular movements and blink artifacts. Note that the data were baseline-corrected before ICA to ensure reliable separation of TMS-evoked data into independent components; due to the non-stationary nature of event-related EEG data, centering over the whole epoch time window could lead to spurious correlations between the latent components, and thus, hinder the performance of ICA (Hernandez-Pavon et al., 2022).
7. Since the removal of ocular artifacts may lead to changes in the baseline, the baseline correction was redone after ICA.
8. The SOUND algorithm (Mutanen et al., 2018) was used to detect and suppress noise from extracranial sources and to interpolate the missing signals in the rejected channels in step 3. The SOUND algorithm utilized the three-layer spherical head model with theoretical 10–20 channel locations (Mutanen et al., 2018, 2020) and the regularization parameter was set to *λ* = 0.1 (Mutanen et al., 2018).
9. Remaining TMS-evoked muscle artifacts were suppressed with the SSP–SIR approach (Mutanen et al., 2016). The SSP–SIR algorithm utilized the three-layer spherical head model with theoretical 10–20 channel locations (Mutanen et al., 2016, 2020) and the two singular vectors spanning most of the artifact subspace were identified as artifactual (Mutanen et al., 2016).
10. The data were low-pass filtered using a finite-impulse response filter with a cut-off frequency of 80 Hz to remove high-frequency content of the signal, which is unlikely to be caused by neuronal activity (Delorme and Makeig, 2004). After the low-pass filtering, the data were upsampled to 5,000 Hz.

The ground truth dataset also underwent *post-hoc* preprocessing using an alternative pipeline that did not depend on the SSP–SIR algorithm. This was done to confirm that any observed differences between the ICA and SSP–SIR cleaning were not attributable to bias in preparing the ground truth data. That is, in the primary pipeline, SSP–SIR was used to suppress both the real and the simulated artifacts. The *post-hoc* preprocessing pipeline was the same, except that the use of ICA (step 7) was extended in suppressing also muscle artifacts, which were originally handled by the SSP–SIR (step 9), respectively. The independent components corresponding to TMS-evoked muscle artifacts were detected using the automated algorithm (Rogasch et al., 2017). In total, two components were detected as TMS-evoked muscle artifact.

In addition to the simulations, we analyzed open-source TMS–EEG data (Hussain et al., 2019) to characterize real-world muscle artifacts and compare their properties with the simulated artifacts presented here. This study was approved by the National Institutes of Health Combined Neuroscience Section IRB, and all subjects provided their written informed consent before participating. The open-source data were collected from 20 healthy individuals who received 600 monophasic single TMS pulses to the right primary motor cortex, specifically targeting the FDI representation area, at 120% of their motor threshold intensity. The TMS (MagStim 2002, MagStim Co. Ltd., United Kingdom) was directed using neuronavigation (BrainSight, Rogue Research, Montreal) with a TMS-compatible 30-channel EEG system (BrainAmp MR+, Brain Vision). This dataset was selected for analysis because the stimulation location matched that of our ground truth data, and the relatively high stimulation intensity ensured the presence of muscle artifact contamination in most datasets when appropriate preprocessing steps to suppress these artifacts were not implemented. The real-world muscle artifact data were processed using the same preprocessing pipeline as the ground truth data, with the exception that steps 8–10 were omitted to maintain a favorable artifact-to-brain signal ratio. In addition, since the EEG data in this case were recorded without the sample-and-hold amplifier (Virtanen et al., 1999), the time interval (−1 to 5 ms) containing the TMS-pulse artifact was replaced with zeros after preprocessing step 1. Robust detrending should not cause filtering artifacts around the rejected TMS-pulse-artifact interval, as it inherently ignores data segments with abrupt changes in signals when modeling baseline drifts. Additionally, we ensured this through visual inspection. Following the preprocessing, the rejected time interval was interpolated using shape-preserving piecewise cubic interpolation for illustration purposes. To ensure a high artifact-to-brain signal ratio, subjects exhibiting TMS-evoked muscle artifacts with a maximum peak-to-peak amplitude of less than 250 μV were excluded. This resulted in 17 datasets being retained for the artifact characterization analysis. At the end of preprocessing, the first 300 accepted trials were retained from each dataset to balance the dataset sizes. For a detailed description of the data collection of the open-source data, please refer to the original publication (Hussain et al., 2019).

### Muscle-artifact simulation

In this study, we conducted simulations to replicate TMS-evoked muscle artifacts. Each artifact dataset comprised 1,000 data points, sampled at a rate of 5,000 Hz, encompassing the temporal range of −50 to 150 ms relative to the TMS pulse initiation. A total of 173 random trials were included in the artifact dataset. The temporal profiles were modeled using Daubechies order-4 wavelets at scale 4. The Daubechies wavelets were chosen due to their similarity to real muscle artifacts observed in TMS–EEG data (Mutanen et al., 2013). As these simulated wavelets have sharp time courses, they also exhibited a broad-band frequency response comparable to actual muscle artifacts when analyzed with classic Fourier-based methods (Mutanen et al., 2016). This property is important when estimating artifact topographies from high-pass filtered data (cut-off 100 Hz) as done in SSP–SIR. The onset of the artifact was aligned with the initiation of the TMS pulse. To introduce variability across trials, we used a similar approach as introduced in Atti et al. (2023): the parameter *α* was manipulated, ranging from 0 to 1 (see Eq. 13). A value of 0 indicated consistent phase across trials, while *α* = 1 produced trial time courses with completely randomized phase (maximal trial variability).

**Figure 1:**
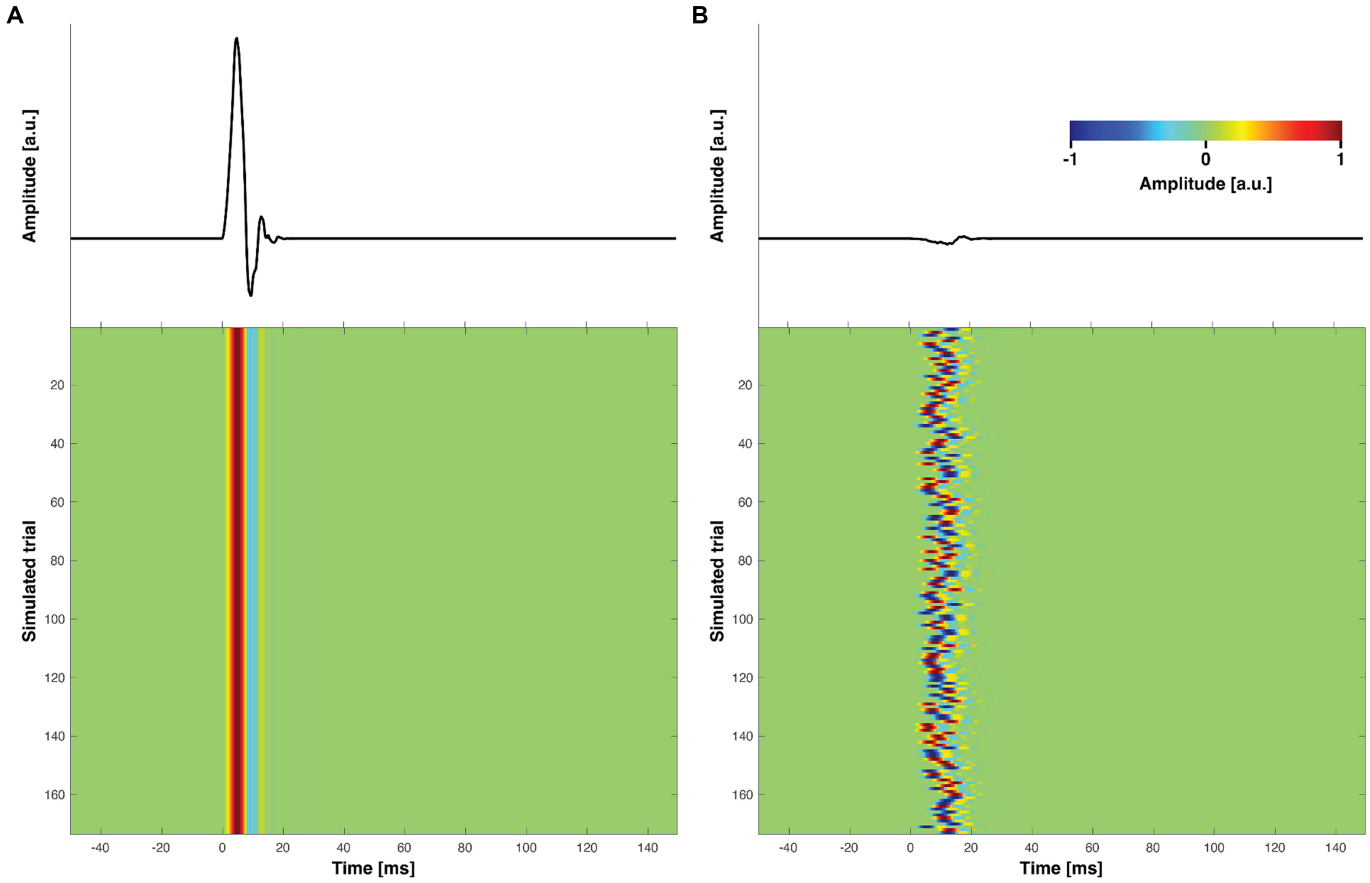
Representative examples of simulated muscle-artifact time courses. **(A)** Muscle-artifact time courses with perfect inter-trial consistency (no inter-trial variability *α* = 0). The top panel displays the mean muscle artifact time course, while the color-coded bottom panel illustrates the variations across trials. **(B)** Muscle-artifact time courses with maximal inter-trial variability (*α* = 1). Similar to **A**, the top panel displays the mean muscle artifact time course, and the color-coded bottom panel depicts the variations across trials. Due to the high inter-trial variability, the amplitude of the average signal in **B** is significantly lower than in **A**. All the amplitudes shown in the figure are in arbitrary units (a.u.).

The artifact time course $a_{i}\left(t\right)$ for a trial *i* can be written as:


(13) $$a_{i}\left(t\right)=\left(1-\alpha \right)\phi_{s}\left(t\right)+\alpha S_{i}\phi_{s}\left(t-\varphi_{i}\right)\text{,}$$

where *α* is the inter-trial variability index given values from 0 to 1, $\phi_{s}\left(t\right)$ is the Daubechies order-4 wavelet, at scale 4, $S_{i}$ is the random sign with values 1 or −1, and $\varphi_{i}$ is the trial-specific random translation bounded between 0 and 10 ms. We restricted the random translation to 10 ms to ensure that the simulated artifact deflection remained within the first 20 ms. This is consistent with findings that real TMS-evoked muscle artifacts typically peak within the first 20 ms following a TMS pulse, as characterized in Mutanen et al. (2013). Representative artifact time courses are shown in Figure 1.

**Figure 2:**
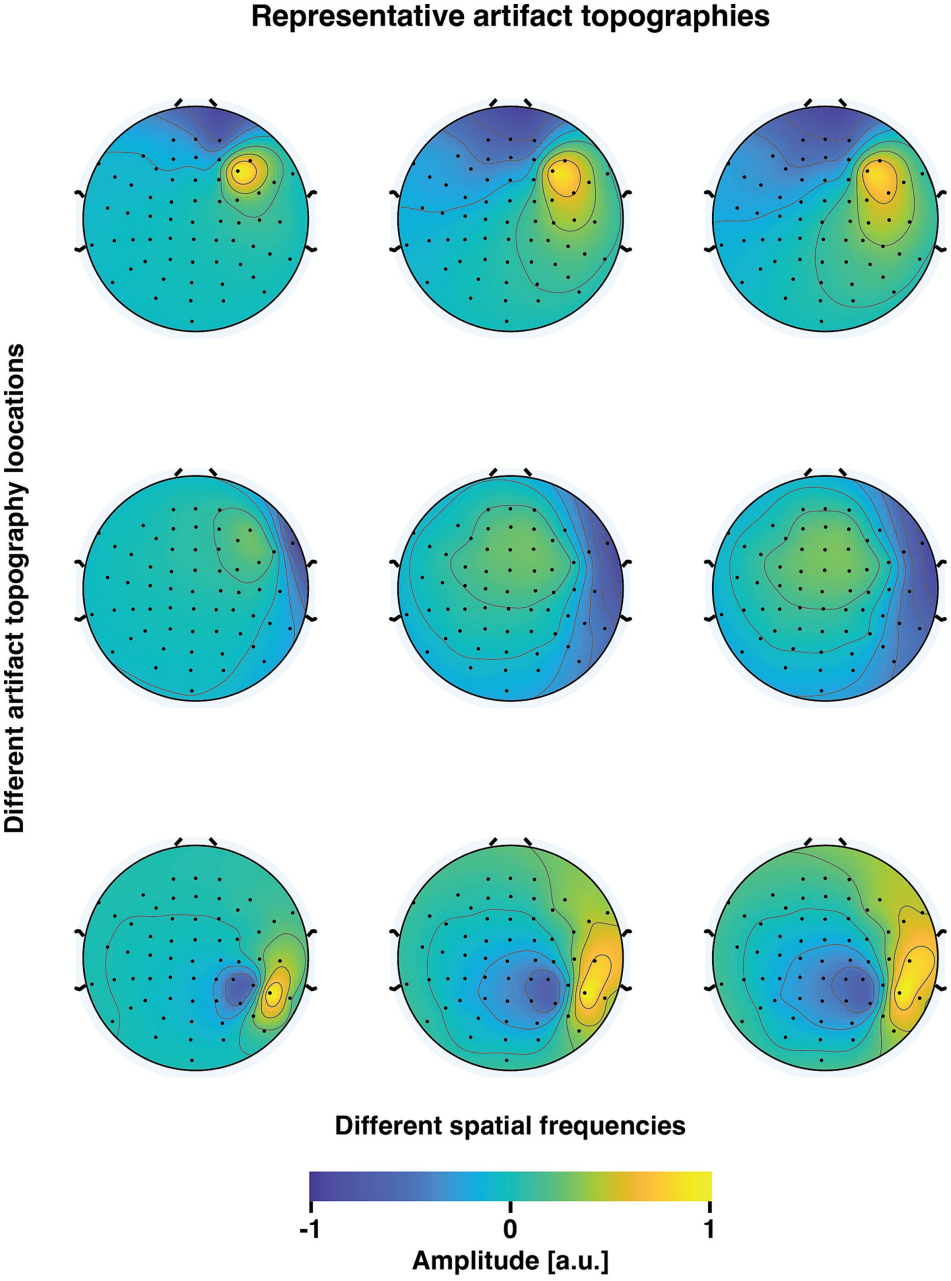
Representative examples of simulated muscle artifact topographies. The three rows correspond to three distinct locations of the muscle artifact potential patterns (selected from a total of 9 drawn patterns). The three columns depict variations in the skull-skin conductivity ratio (0, 100, and 200 in these examples). As the conductivity ratio increases, there is a gradual decrease in the spatial frequency of the topographies. Consequently, this increases the similarity between the artifact topographies and the underlying neuronal potential patterns.

To extend the muscle artifact to a multidimensional signal consisting of 60 channels, we multiplied the trial-specific time courses $a_{i}\left(t\right)$ with a simulated muscle artifact topography. We simulated 9 different artifact topographies, all located on the right side of the head, as the stimulation of right M1 is likely to activate mainly the right lateral scalp muscles (Mutanen et al., 2013). The topographies were drawn from an anatomical head-model-based lead field of the subject, from sources within the right hemisphere. This placement of cortical current sources yielded bipolar lateral potential patterns akin to those typically associated with real muscle artifacts. To simulate artifact topographies with different spatial frequencies, we used different conductivity contrasts between the skull and the skin/brain. In total, 11 different skull-to-skin/brain-conductivity contrasts were tested; 1, 1/20, 1/40, 1/60, 1/80, 1/100, 1/120, 1/140, 1/160, 1/180, 1/200. Consequently, a spectrum of realistic topographies emerged, with varying degrees of aligning with the underlying ground truth neuronal topographies (see Figure 2 for examples). The alignment was measured in terms of the angle (°) between the 60-dimensional artifact and neuronal signal vectors (topographies). We calculated the angle between each variant of the artifact topography and the ground truth TEP topographies at each time point within the first 100 ms following the TMS pulse. Next, we determined the minimum angle within this time interval by calculating the 5th percentile across the studied time points. Finally, since there were nine unique artifact topographies for each skull–skin contrast, from which the artifact topography was sampled for each simulation run, we computed the median of the minimum angles.

It is important to note that the head model employed was not specifically designed to accurately mimic the intricate physiology of scalp muscle fibers. The MRI-based three-layer head model was utilized purely as a phenomenological model to create muscle artifact topographies that exhibited varying degrees of correlation with the underlying neuronal potential patterns.

**Figure 3:**
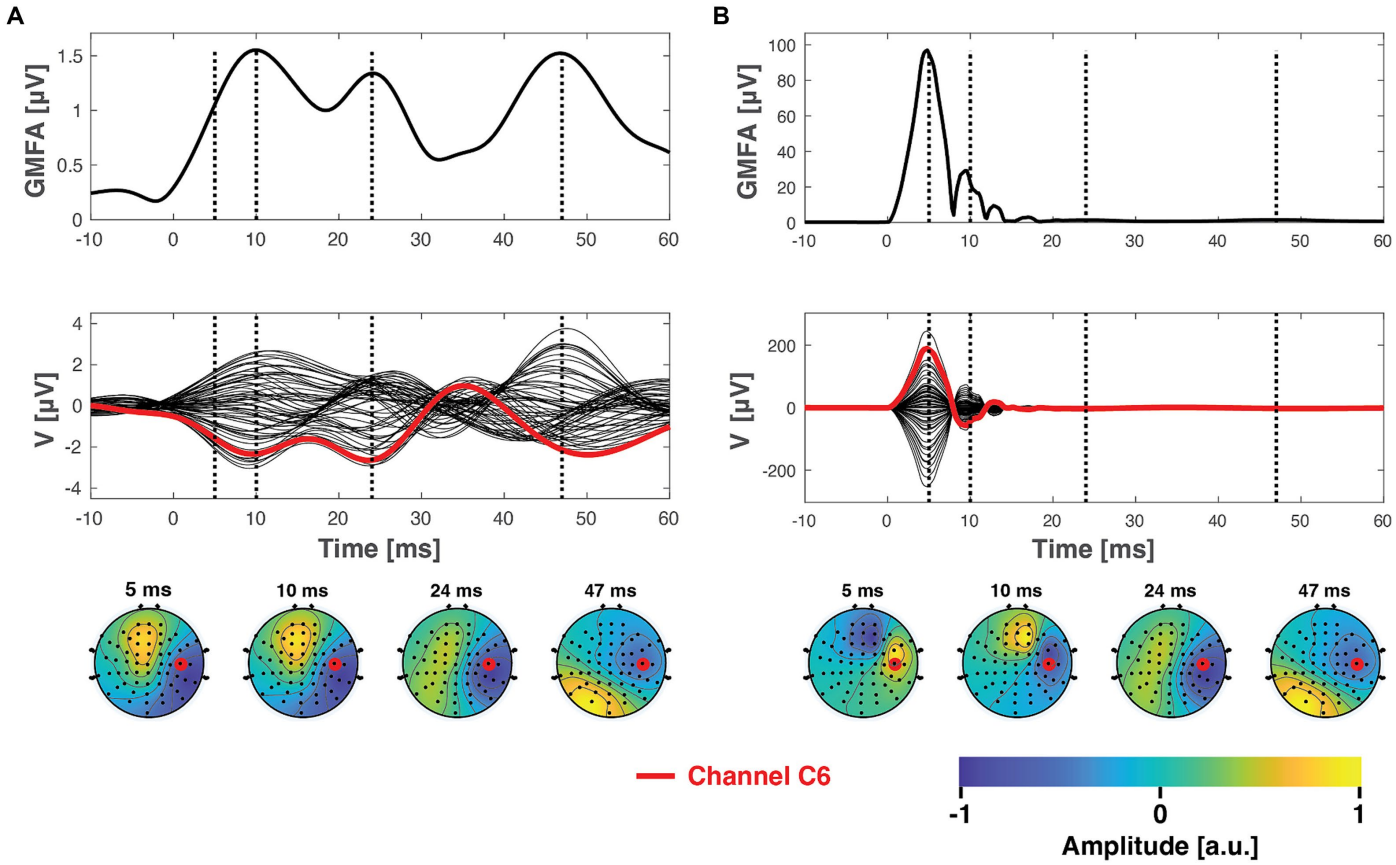
The ground truth TMS–EEG dataset before **(A)** and after **(B)** superimposing the simulated artifact signals. The top panel displays the global mean field amplitude (Lehmann and Skrandies, 1980) of the average TMS–EEG responses. The central panels depict the mean time courses from all 60 channels in a butterfly plot. The bottom panels present topographies at specific latencies, indicated by the dashed vertical lines. Here, the chosen artifact signals represent a scenario with perfect inter-trial consistency (no inter-trial variability α=0 ) and an artifact topography that possesses the highest spatial frequency (indicating the smallest similarity to the underlying ground truth signals).

The ground truth dataset is visualized in Figure 3A, which also illustrates the data after adding simulated artifacts to the dataset (Figure 3B). As seen in Figure 3, before the adding the artifact data, consisting of the trial time courses and topography, the artifact data was scaled to result in maximum peak-to-peak amplitudes of approximately 250 μV. The effect of artifact amplitude on the effectiveness of artifact suppression was also tested under both low and high amplitude artifact conditions, with peak-to-peak amplitudes of 50 μV and 1 mV, respectively.

### Data analysis

We simulated 121 distinct artifact types, derived from the combination of 11 unique topographical conditions, with varying degrees of similarity with the neuronal topographies, and 11 different inter-trial-variability scenarios. For every artifact type, 100 random representations were generated. Each representation consisted of 60 channels, 1,000 time points, and 173 trials. During each simulation, the topography was selected at random from a predetermined subset of 9 lateral, tangential current sources, with a predefined contrast between the skull and skin. Although the set α -value ensured that trial time courses exhibited roughly analogous inter-trial variation, the precise time courses for each trial differed with every simulation run. The simulated artifact representations were superposed on the ground truth TMS–EEG dataset. Subsequently, the dataset containing these artifacts was processed using either the ICA or the SSP–SIR algorithm. No other preprocessing methods were used at this stage.

For the ICA processing, trials were concatenated, and the FastICA algorithm was employed to decompose the dataset into 60 independent components (Hyvärinen and Oja, 2000). We utilized the symmetric approach in combination with the tanh contrast function. To retain the objectivity of the data-cleaning approach, we allowed the ICA to function optimally; we subtracted the independent component from the artifactual dataset that exhibited a topography most closely resembling the true simulated artifact potential pattern.

The three-dimensional artifactual dataset was also processed separately with SSP–SIR. As the simulated artifacts did not average well in such studied scenarios where time-locking to the TMS pulse was low, each trial was cleaned independently with SSP–SIR. In the SIR step, we utilized the three-layer spherical head model with theoretical 10–20 channel locations (Mutanen et al., 2016, 2020). That is, a different head model was used for SSP–SIR than the one used for simulating the artifact topographies. SSP–SIR identified artifact topographies from data high-pass filtered at a cutoff of 100 Hz, utilizing a time window of −10 to 30 ms relative to TMS onset. To allow SSP–SIR to function optimally, we projected out the signal dimension, demonstrating the best correlation with the known simulated artifact topography. As implemented in the open source data-analysis tools (Mutanen et al., 2020), SSP–SIR was set to suppress artifact topographies only during the early time window of −10 to 30 ms. The transition between the suppressed and untouched data was smoothened with a 10 ms median filter.

We quantified the cleaning performance of ICA and SSP–SIR by calculating the mean relative error (RE) between the true ground truth and the uncovered neuronal signals (Eq. 14). This calculation was performed by comparing the cleaned dataset with the ground truth signal for each trial over a time interval from 0 to 50 ms, and then averaging the trial-specific relative error scores:


(14) $$RE=\frac{1}{N_{t}}\overset{N_{t}}{\underset{i=1}{\sum }}\overset{N_{c}}{\underset{c=1}{\sum }}\overset{50}{\underset{t=0}{\int }}\frac{{\left({\tilde{y}}_{c}^{i}\left(t\right)-y_{c}^{i}\left(t\right)\right)}^{2}}{y_{c}^{i}{\left(t\right)}^{2}}dt\text{,}$$

where ${\tilde{y}}_{c}^{i}\left(t\right)$ and $y_{c}^{i}\left(t\right)$ are the cleaned and the ground-truth signals measured at trial *i,* at time *t,* in channel *c*, respectively, and *N_t_* and *N_c_* are the number of trials and channels, respectively. Later time intervals were not included in the main analysis because of the way the SSP–SIR correction was implemented; any responses later than 50 ms should be left completely unaltered, and thus, SSP–SIR should trivially perform better than ICA. However, we confirmed this prediction in an additional analysis by quantifying RE using the time interval 50–100 ms, where the maximum amplitude of the simulated artifact was set to ~250 μV.

By choosing to average these trial-specific scores instead of directly quantifying the relative error between the average TEPs, we minimized the potential bias in the RE metric due to the inter-trial variability of the artifact. The chosen approach ensured that the conditions of large inter-trial variability, which hinder the effective averaging of simulated artifacts (as illustrated in Figure 1), do not skew the RE metric. To statistically evaluate the impact of trial variability and topographical similarity on the efficacy of the cleaning methods, we conducted a two-way ANOVA on log-transformed RE, the topographical similarity and inter-trial variability as factors. ANOVA was applied separately for ICA and SSP–SIR. Before running ANOVA, the transformed data were inspected visually with histograms and box plots for approximate normality and homoscedasticity. Because the ANOVA test was run in all three tested artifact-amplitude conditions, the *p*-values were corrected for multiple comparison with the Bonferroni method.

Finally, we characterized the properties of real-world TMS-evoked muscle artifacts, analyzing an additional artifactual TMS–EEG datasets. This was done to assess which simulated artifact variants most closely resembled real-world muscle artifacts. To assure that signal space angles in real-world data were comparable to those in the simulation analysis, it was crucial to transform the real-world data into the same 60-dimensional sensor space corresponding to the ground truth and simulated artifact data. We employed the minimum-norm estimation (MNE)-based extrapolation technique (Ilmoniemi and Numminen, 1992), widely utilized in previous MEG literature (Numminen et al., 1995; Burghoff et al., 2000; Wang and Oertel, 2000; Wübbeler et al., 2001; Knösche, 2002; Ross et al., 2011; Marhl et al., 2022). This approach was chosen due to its alignment with the principles of electromagnetic modeling in EEG signals, as opposed to the common practice of spline surface interpolation. Unlike spline interpolation, which can violate Maxwell’s equations, MNE interpolation ensures compatibility with the known or assumed source volume and head geometry, thereby producing EEG patterns that accurately reflect the underlying physiological processes. In MNE, we employed a spherical three-layer head model (Mutanen et al., 2016, 2018). To prevent MNE extrapolation from attenuating the original signals, we utilized a very low regularization level in the Tikhonov-regularized MNE of $\lambda =10^{-5}\;\mathrm{trace}\;\left(LL^{T}\right)$ , where **L** represents the spherical head-model-based lead field matrix. To concentrate the analysis on the muscle artifact signals, TEPs before the SOUND step, from the time interval 0 to 50 ms after the TMS pulse, were separated into component using singular value decomposition. $Y_{0-50\mathrm{ms}}=U\Sigma V^{T}$ . The first column vector of the U matrix was identified as the muscle artifact topography. The corresponding artifact time courses for trials *k* could be computed as $S_{1}^{k}=U_{1}^{T}Y^{k}$ . In this analysis, the artifact was extracted directly from the TEP datasets without employing the same high-pass filtering step as in the SSP–SIR procedure, to avoid introducing a bias in favor of SSP–SIR. Given that the muscle artifacts present in the original data were substantial, measured in hundreds to thousands of microvolts, simply isolating the first singular component from the original dataset should ensure a considerably high artifact-to-brain signal ratio for the subsequent artifact characterization process. We evaluated the inter-trial variability of real muscle artifacts by computing the inter-trial coherence (ITC) (or phase-locking factor) (Tallon-Baudry et al., 1996) within the estimated artifact time courses. Note that the ITC values have an opposite convention compared to our inter-trial variability index *α*; ITC = 1 means perfect inter-trial phase alignment whereas *α* = 1 indicates fully random trial-specific phases. Furthermore, we estimated the similarity between the artifactual and neuronal topographies. To improve the signal-to-noise ratio of the neuronal topographies, the data were passed through the SOUND algorithm. We then computed the angle between the estimated artifact topography $U_{1}$ and the potential patterns at each trial and time point. Without the SOUND filtering, the angle between the muscle-artifact topography and the rest of the EEG would likely have captured, at least partially, angles related to plain recording noise. Furthermore, the utilization of SOUND here also enhanced the direct comparability between the results obtained with the real-world artifactual data and the analysis addressing the simulated artifacts and ground truth data.

## Results

**Figure 4:**
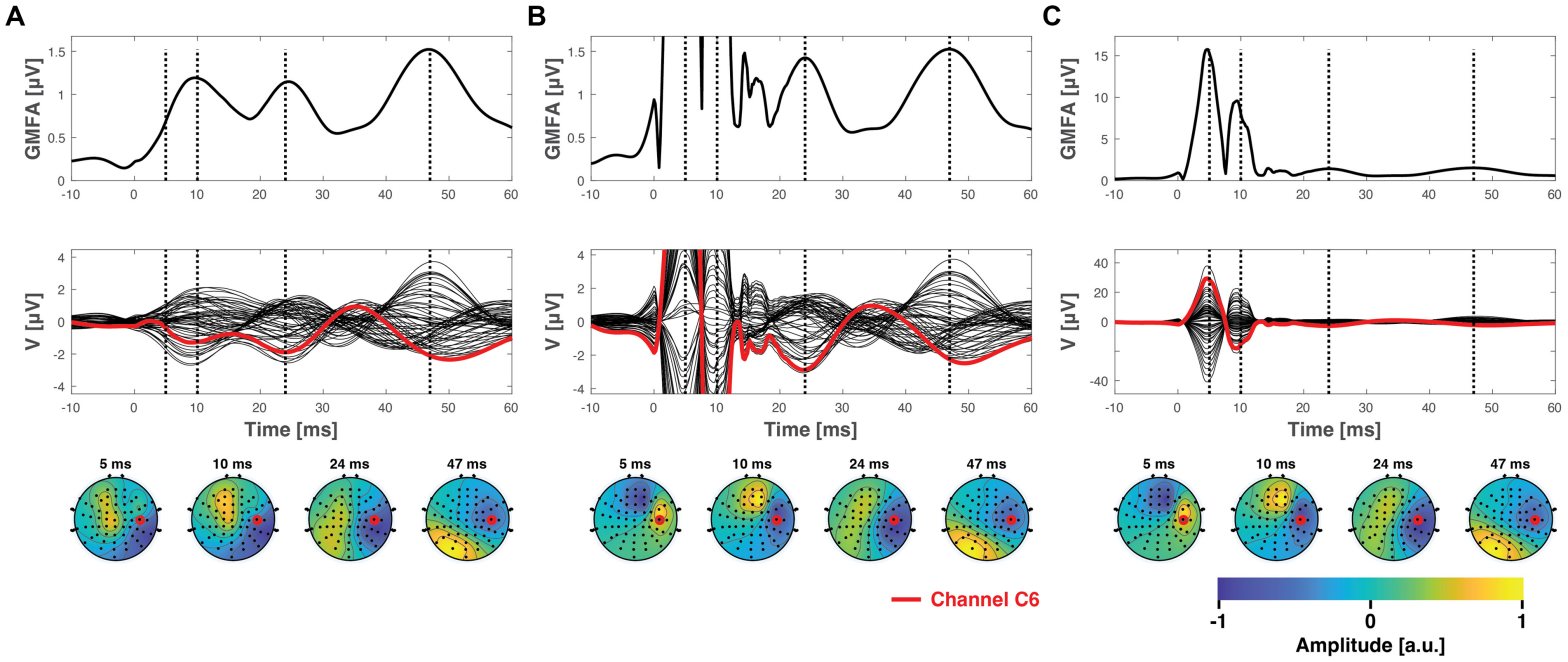
The ground truth data, presented in Figure 3A, after being superposed with simulated artifacts (see Figure 3B) and subsequently cleaned using SSP–SIR **(A)** or ICA **(B,C)**. **(B)** Shows the ICA-corrected signals in the same *y*-axes with the SSP–SIR-corrected data for direct comparison, whereas panel **C** shows the full extent of the ICA results. SSP–SIR can recover especially the global mean field amplitude and channel time courses well in ideal conditions. The topographies are also recovered adequately, albeit they suffer from mild attenuation due to spatial filtering. ICA was able to clearly attenuate even the time-locked artifacts, but there are still clear residual artifact signals left. ICA performed much better in suppressing artifacts that varied across the trials (inter-trial variability α≈1 ) compared to the visualized condition, (inter-trial variability α=0 ), but as the variable artifacts do not average well, visualizing them as average butterfly plots and GMFA curve is not informative.

**Figure 5:**
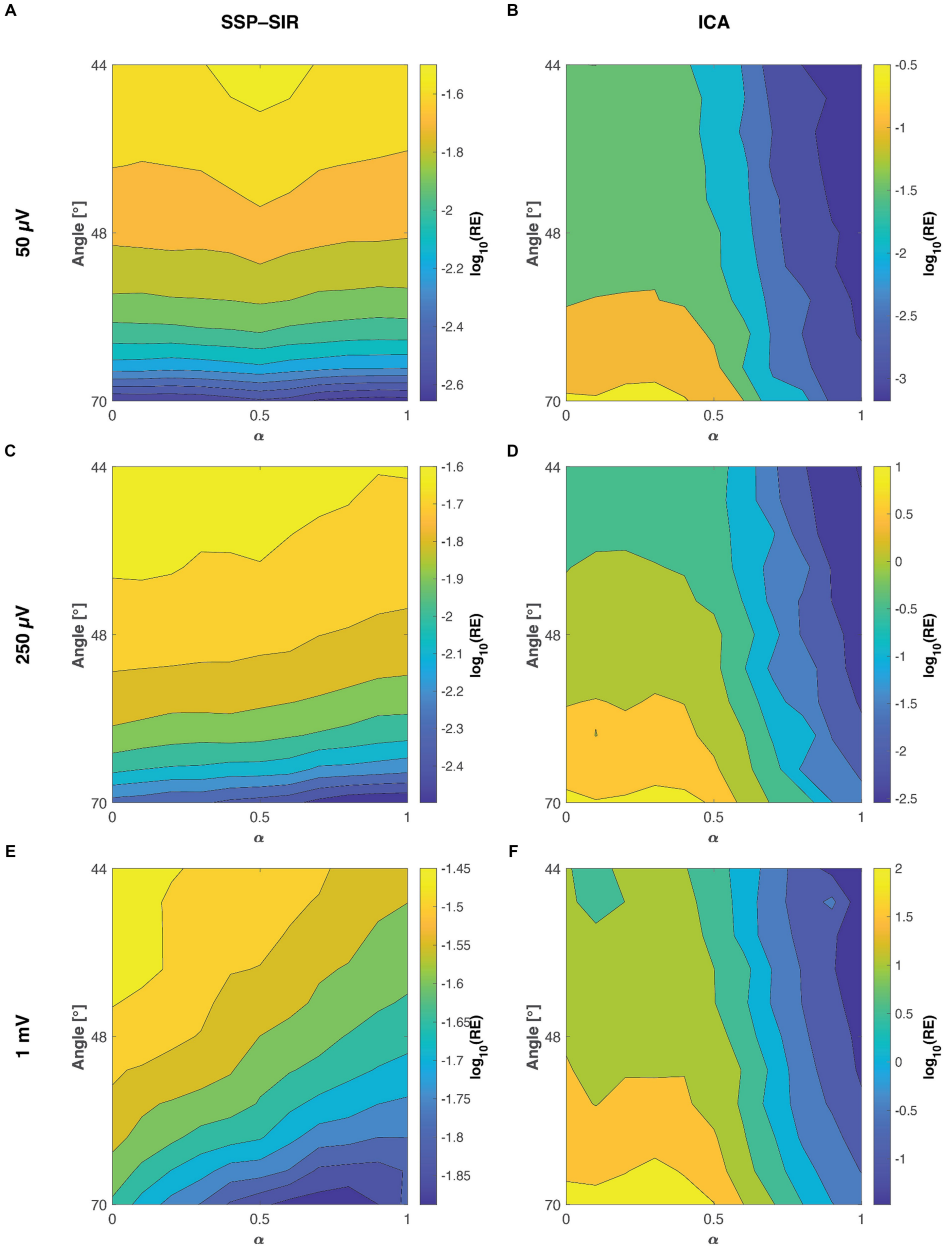
The mean relative error (RE) between the true ground truth signal and the recovered TMS–EEG responses at the three different tested artifact amplitudes as a function of the signal-space angle between the artifact and neuronal topographies and the inter-trial variability ( α ). The smaller the angle between the artifact and neuronal topographies, the more similar they are. Panels **A,C,E**, show the performances of SSP–SIR when rejecting a 50 μV, 250 μV, or 1 mV amplitude artifact, respectively. Panels **B,D,F** show the corresponding results for ICA. To provide a clear illustration of the variability in results, we applied a log10 transformation to the relative error values. For instance, values of 1, 0, and −1 here correspond to relative errors of 1,000, 100, and 10%, respectively. Note that the colormap scales for different panels differ; the RE of SSP–SIR is overall lower than that of ICA.

Both the ICA and SSP–SIR methods demonstrated efficacy in removing artifacts under favorable conditions. Figure 4 provides a visual representation of the cleaning results for an example dataset. Figure 5 depicts the relative error due to artifact rejection within the 0–50 ms time interval after the TMS pulse, illustrating the varying sensitivities of ICA and SSP–SIR across different artifact conditions. The figure clearly demonstrates the differential responses of the two methods to specific artifact characteristics; SSP–SIR was particularly sensitive to the shape of the artifact topography, whereas the performance of ICA depended more strongly on the inter-trial variability of the artifact.

As predicted, SSP–SIR resulted in very small RE for the time interval 50–100 ms after the TMS pulse, ranging from 0.06 to 0.18%. However, ICA also performed well within this time interval, yielding RE values of just 0.4 to 1.6%. This suggests that with ICA the possible overcorrection was mainly emphasized in the early time interval (data not shown).

The overall results remained consistent when the preprocessing of the ground truth data relied on ICA instead of SSP–SIR for muscle artifact rejection (data not shown). This suggests that the use of SSP–SIR in the preprocessing of the ground truth data did not introduce bias in favor of SSP–SIR in the simulations.

We simulated artifact topographies as cortical current sources using a three-layer head model with varying skull conductivities. This phenomenological model produced artifact topographies that exhibited varying degrees of similarity to the underlying neuronal potential patterns. Because the neuronal activity fluctuates over time, most of the ground truth topographies in the first 100 ms following the TMS pulse demonstrated only weak correlations with the simulated artifact topography. However, among the top 5% most correlated time samples, the signal space angle between the artifact and neuronal signals ranged between 70° and 44°. This was sufficient to introduce clear trends in the simulations, revealing particularly the sensitivity of SSP–SIR on the topographical congruence between the artifact and neuronal topographies (see Figure 5).

### ICA is particularly sensitive to inter-trial variability

As clearly demonstrated in Figure 5, ICA displayed a heightened sensitivity to inter-trial variability. Additionally, the topographical shape of the artifacts also influenced the cleaning performance to an extent. The results from the two-way ANOVA confirmed that both inter-trial variability and topographical similarity significantly influenced the relative error between the cleaned and the ground truth signal, with *p*-values less than 0.001. However, when examining the effect sizes, inter-trial variability predominantly accounted for the observed variance with a notable effect size, whereas topographical similarity had a substantially lower effect size (see Table 1 for all the effect sizes and *p*-values). Importantly, our analysis did not reveal any significant interaction between the topographical and trial-variability characteristics. The amplitude of the artifact did not affect the overall trend of the results (see Figure 5 and Table 1), although expectedly, RE overall increased with the amplitude of the artifact.

**Table 1:** The ANOVA results for the tested artifact amplitudes.

Artifact amplitude	SSP–SIR		ICA	
	Inter-trial variability	Topographical similarity	Inter-trial variability	Topographical similarity
50 μV	*p* < 0.001 *η*^2^ = 0.004	*p* < 0.001 *η*^2^ = 0.105	*p* < 0.001 *η*^2^ = 0.652	*p* < 0.001 *η*^2^ = 0.002
250 μV	*p* < 0.001 *η*^2^ = 0.012	*p* < 0.001 *η*^2^ = 0.057	*p* < 0.001 *η*^2^ = 0.536	*p* < 0.001 *η*^2^ = 0.002
1 mV	*p* < 0.001 *η*^2^ = 0.043	*p* < 0.001 *η*^2^ = 0.115	*p* < 0.001 *η*^2^ = 0.516	*p* < 0.001 *η*^2^ = 0.002

### The performance of SSP–SIR depends strongly on the shapes of the artifact topographies

SSP–SIR exhibited a pronounced sensitivity to topographical similarity (see Figure 5). However, inter-trial variability also played a role in determining the cleaning outcome. The two-way ANOVA results mirrored this observation, indicating that both inter-trial variability and topographical similarity significantly influenced the relative error when compared to the ground truth signal (see Table 1). In terms of effect sizes, topographical similarity emerged as the dominant factor as opposed to the inter-trial variability (Table 1). Like in the ICA analysis, no significant interaction was detected between the two studied artifact properties. However, the effect sizes overall were much smaller for the SSP–SIR compared to ICA, suggesting more consistent performance for the SSP–SIR across the studied conditions. The amplitude of the simulated artifact did not change the nature of the statistical results. However, as with ICA, RE overall increased with the amplitude of the artifact. Furthermore, with the very large artifact (1 mV peak-to-peak-amplitude) also the inter-trial variability started to play a more significant role (see Figure 5 and Table 1).

### Real TMS-evoked muscle artifacts are strongly time-locked, and their topographies correlate only weakly with neuronal potential patterns

**Figure 6:**
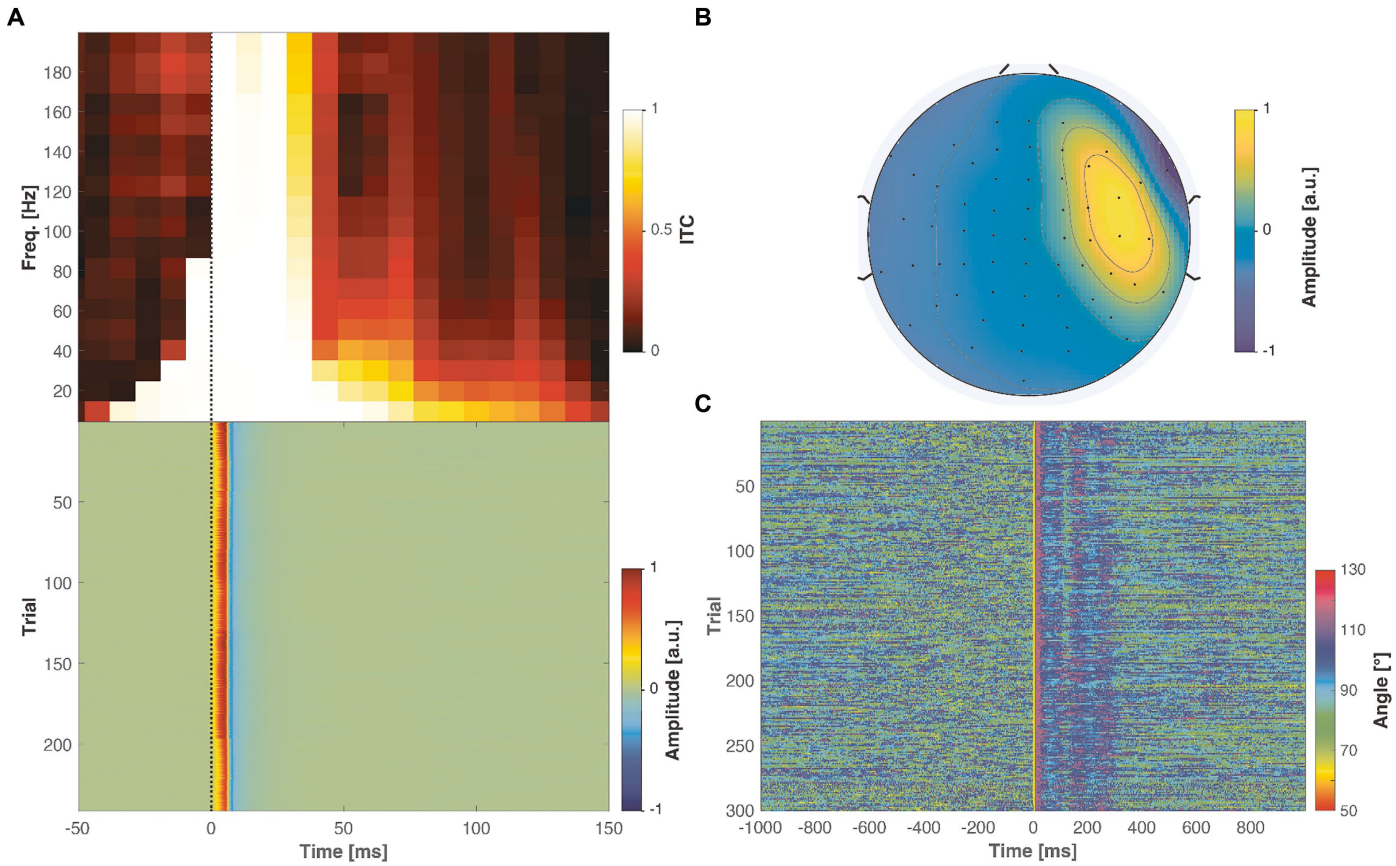
Properties of real muscle artifacts in an example real-world artifactual dataset. **(A)** Inter-trial coherence (ITC) shows a very strong consistency across the trials (top panel), reflected in the biphasic trial responses (bottom panel). **(B)** The muscle artifact created a bipolar topography, emphasized in the right lateral channels. **(C)** When quantifying the similarity between the artifact topography and the neuronal potential patterns across the measured trials and time span, a vast majority of the angles vary between 80 to 100°, which is larger compared to any of the simulated cases (see Figure 5).

**Figure 7:**
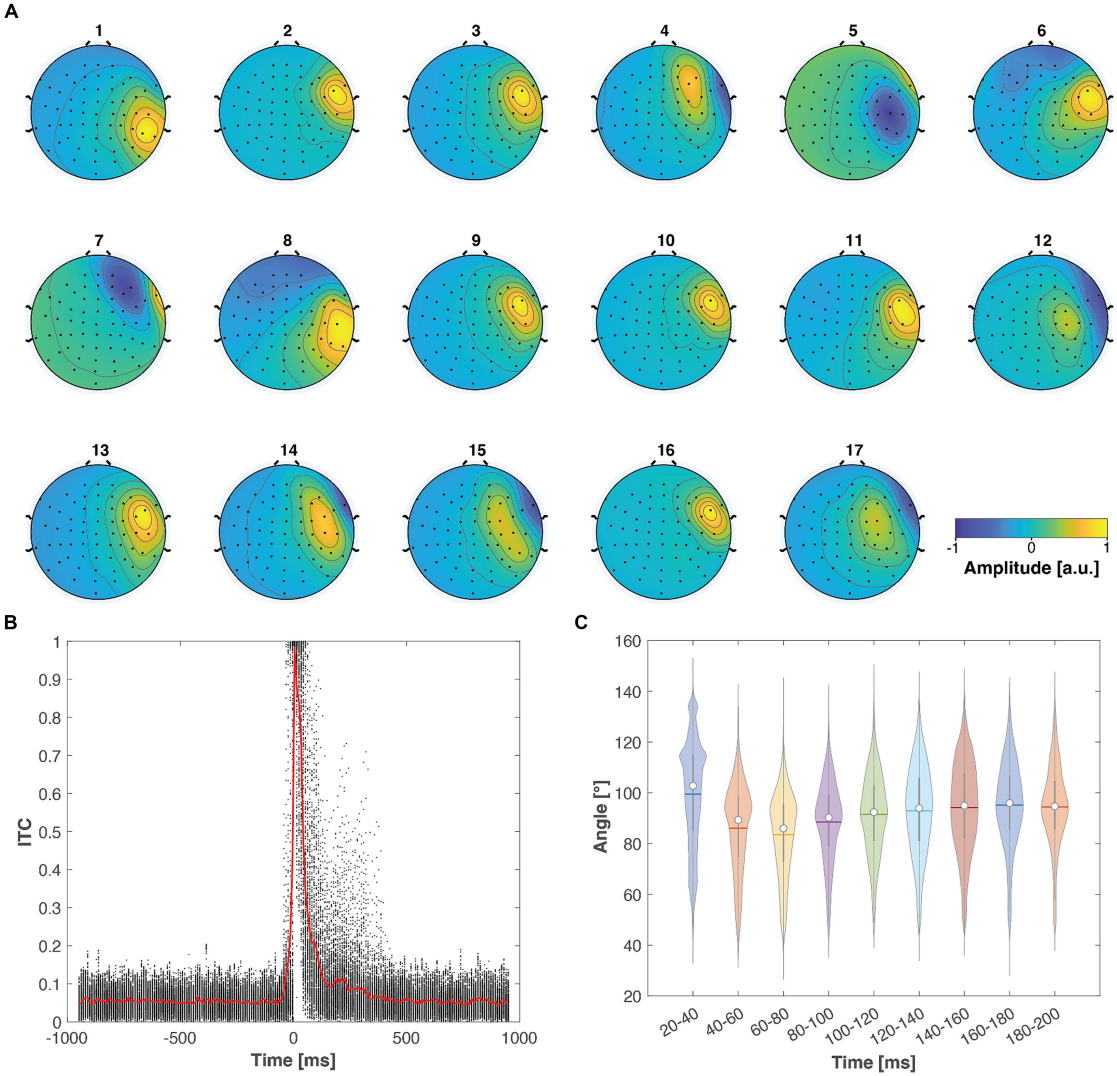
TMS-evoked muscle artifacts demonstrated consistent characteristics across the 17 studied real-world datasets. **(A)** Across all datasets, the artifact exhibited a focal topography, particularly emphasized in the right lateral channels. **(B)** The scatter plot illustrates the ITC values across subjects and frequencies (see Figure 6A), with the red solid line indicating the mean ITC as a function of time. The muscle artifacts consistently showed very strong ITC values immediately following the TMS pulse. **(C)** The violin plots display the distribution of the angle between the artifact topography and neuronal potential patterns across subjects and trials. The white dots, horizontal lines, and vertical lines represent the median, mean, and lower and upper whiskers, respectively. These violin plots indicate that most of the studied artifact–neural topography angles predominantly lie between 80° and 100°.

All in all, the analysis of the real-world artifactual data demonstrates that TMS-evoked muscle artifacts are strongly time-locked and manifest themselves in EEG as lateralized topographies, which differ substantially from the neuronal potential patterns. An example of a real-world muscle artifact is illustrated in Figure 6. In the time domain, the muscle artifact signals were seen as early biphasic responses that were highly replicable and consistent across the trials. This high consistency resulted in ITC values near one, indicating perfect phase locking. On the other hand, the artifact topography differed substantially from the other EEG potential patterns. Based on the inspection of real-life muscle artifacts, the most realistic scenario in the simulated cases is the one with maximum angle between the artifact and ground truth neuronal topographies of 70° and close to minimal inter-trial variability α≈0 . The results obtained from all the studied artifactual TMS–EEG datasets are summarized in Figure 7.

## Discussion

We analyzed how the spatiotemporal attributes of to-be-rejected artifacts determine the data-cleaning capacities of ICA and SSP–SIR. Through the simulations, where distinct TMS-evoked artifacts were superposed on a genuine TMS–EEG dataset, we could quantitatively compare the uncovered signals with the ground truth. The results offer insights into the differential sensitivities of ICA and SSP–SIR. Our findings suggest that ICA functions optimally when the artifacts show pronounced inter-trial variability. This observation confirmed our initial hypothesis, affirming that ICA can separate artifacts that are relatively independent of time-locked TMS-evoked activity. Conversely, SSP–SIR is sensitive to the topographical congruence between the artifact and neuronal signals, but insensitive to the inter-trial variability, validating our initial expectations. Regardless of the artifact amplitude, ICA primarily showed sensitivity to inter-trial variability, while the performance of SSP–SIR largely depended on the topographical shape of the artifact. As expected, both methods demonstrated improved performance with lower artifact amplitudes. The amplitude-simulation results mirror our practical experience: suppressing artifacts of a few hundred microvolts is often feasible, but the potential for artifact suppression rapidly diminishes as the amplitude approaches the millivolt scale.

SSP–SIR exhibited increased sensitivity to inter-trial variability when dealing with high artifact amplitudes. This heightened sensitivity can be traced back to the calculation process of RE. When the inter-trial variability of artifacts was minimized (set to 0), any remaining artifacts were most noticeable around 6 ms. This coincided with instances where the ground truth signal exhibited relatively low amplitudes (as illustrated in Figure 3), leading to larger RE values. Conversely, when inter-trial variability was high, residual artifacts also appeared around 10 ms in some trials. This occurred precisely when the ground true signal reached its maximum GMFA (1.5 μV). As a result, the RE values in those trials were comparatively lower. Thus, when averaging the RE values across trials, the overall RE slightly decreased under conditions of high inter-trial variability.

While the estimated effect sizes aligned with our hypotheses, they highlighted considerable differences between the two tested methods. Notably, the effect sizes indicated that the influence of variation in different artifact properties was smaller for SSP–SIR than it was for ICA, which was very strongly influenced by variation in the inter-trial variability of the artifact. Irrespective of the varied artifact parameters, SSP–SIR maintained a more uniform performance in revealing the underlying ground truth with small relative error values overall. A critical assumption underlying SSP–SIR is that muscle-artifact signals have a consistent topography across the different temporal frequencies. The simulations here always maintained this consistency, which could have potentially skewed the results in favor of SSP–SIR. When applying SSP–SIR in real world, it is often necessary to reject several artifact components, possibly due to slight variations in the artifact topography as a function of temporal frequency.

In theory, the ground truth data could contain some real residual artifacts, which, if correctly suppressed, might be mistakenly interpreted as overcorrection of the “neuronal” ground truth data. To mitigate this risk, we deliberately chose a high-quality dataset for our ground truth, ensuring minimal residual artifacts. Furthermore, closer inspection of individual simulation runs revealed that variations in cleaning performance were in most cases attributable to the limitations of SSP–SIR or ICA in effectively suppressing simulated artifacts in suboptimal conditions, rather than to the excessive suppression of the ground truth data.

To answer which method is optimal for muscle-artifact suppression requires a thorough understanding of the properties of such artifacts. The real-world artifactual datasets analyzed here suggested that muscle artifacts have both high inter-trial consistency (low inter-trial variability) and low topographical similarity with the neuronal signals, promoting the use of SSP–SIR over ICA. Although the results here strongly support the use of SSP–SIR for rejecting TMS-evoked muscle artifacts, it is important to note that reliable utilization of SSP–SIR always requires that the time-locked neuronal activity of interest is absent in the high-pass filtered data that is used to estimate the time-locked artifact topographies. Here, we assumed that no significant brain activity exists above 100 Hz.

ICA exhibited diminished efficacy in rejecting artifacts with low inter-trial variability. This finding is in line with the results of the work by Atti et al. (2023), which elaborates further the optimal temporal conditions for applying ICA in artifact removal and theory behind ICA. However, for instance, ocular artifacts are characterized by their sporadic occurrence and often lack time-locking to the TMS pulse, which makes them particularly susceptible to ICA cleaning. On the other hand, their widespread frontal topographies and dominance in temporal frequencies below 10 Hz mean that the SSP–SIR approach, as outlined in Mutanen et al. (2016), is ill-suited for handling these types of artifacts. Thus, despite its limitations in cleaning time-locked artifact signals, the obtained results do not question the general usefulness of ICA for rejecting various artifact and noise signals from TMS–EEG datasets.

Figure 5 shows a counterintuitive peculiarity in the results: when the inter-trial variability was low, RE of ICA increased with greater dissimilarity between artifact and brain topographies. In this special case, a more detailed examination of individual simulation runs revealed that when the artifact topography closely resembled the early neuronal topographies, ICA tended to mistakenly blend a portion of the ground truth data with the artifact signal, leading to overcorrection. Conversely, when the artifact topography was more distinct from the underlying ground truth data, it was more common for some residual artifact to persist. Neither outcome is desirable; however, we found that overcorrection typically resulted in lower RE values.

To maintain analytical simplicity, we utilized the *a priori* knowledge from our simulations that the modeled artifact consisted of a single component. This led us to adopt a similar approach as in Atti et al. (2023) where the independent component most closely resembling the simulated artifact was discarded. It is plausible that ICA segmented the artifact into multiple components, potentially enhancing performance had these components been manually identified. However, this approach would have been impractical due to the sheer volume of simulation runs involved. While semi-automated ICA techniques are available today (Rogasch et al., 2017; Pion-Tonachini et al., 2019), they necessitate the adjustment of numerous hyperparameters, which adds complexity to result interpretation. Consequently, we opted against using automated ICA, aiming to maintain simplicity in our analysis.

If a TMS–EEG dataset consists of both blink and early time-locked muscle artifacts, ICA can be used as a separate step, prior to SSP–SIR, to cancel ocular artifacts. Based on these results, we recommend SSP–SIR over ICA for suppressing time-locked artifacts, for instance when early TMS-evoked responses are of particular interest. However, it is important to remember that even SSP–SIR correction is always a compromise, and the underlying brain responses can be attenuated in the process. Mutanen et al. (2022) recommended analyses to quantify the degree of undesired attenuation in the neuronal signals of interest. SSP–SIR is often used in combination with the SOUND algorithm, which is known to also attenuate muscle artifacts. Thus, SOUND may interact with the performance of SSP–SIR in practical preprocessing pipelines. However, comparing the combination of SOUND and SSP–SIR to using SSP–SIR or ICA alone falls outside the scope of this study.

In these simulations, we exclusively varied the inter-trial variability of the phase of the artifact. However, it is conceivable that also the topography of certain artifacts might also fluctuate from trial to trial. Nevertheless, based on our heuristic experience, the topographies of both TMS-evoked muscle artifacts and ocular artifacts tend to be highly replicable across epochs.

To summarize, the choice between ICA and SSP–SIR depends on the specific nature and properties of the artifacts of interest. As researchers continue to utilize advanced signal-processing tools, profound understanding of the strengths and limitations of different methods, as well as the characteristics of different artifact signals, will be crucial in optimizing EEG data cleaning.

## Data availability statement

The ground truth dataset presented in this article is not readily available because of EU-legislation and the GDPR restrictions. To run the simulation code, you can utilize your own pre-processed datasets. Requests to access the datasets should be directed to tuomas.mutanen@aalto.fi. The code will be made available in Github: https://github.com/tuomasmutanen/SSP-SIR_vs_ICA.

## Ethics statement

The studies involving humans were approved by Ethics Committee of the Hospital District of Helsinki and Uusimaa. The studies were conducted in accordance with the local legislation and institutional requirements. The participants provided their written informed consent to participate in this study. Written informed consent was obtained from the individual(s) for the publication of any potentially identifiable images or data included in this article.

## Author contributions

TM: Writing – review & editing, Writing – original draft, Visualization, Validation, Supervision, Methodology, Investigation, Funding acquisition, Formal analysis, Data curation, Conceptualization. II: Writing – review & editing, Writing – original draft, Methodology, Investigation, Conceptualization. IA: Writing – review & editing, Methodology, Investigation, Conceptualization. JM: Writing – review & editing, Supervision, Methodology, Investigation, Conceptualization. RI: Writing – review & editing, Supervision, Resources, Project administration, Funding acquisition, Conceptualization.
